# Dr. Spence, fin the Sesamum

**Published:** 1802-12-01

**Authors:** George Spence

**Affiliations:** Lucea


### To Dr. BRADLEY.

Dear Sir,

THE prevalence of the Dyſentery, in its moſt virulent ſtate, and among all ranks of people in St. Lucea, affords me an opportunity of doing juſtice to the laudable and benevolent intentions of Sir S. Clarke, by communicating the few obſervations I have been able to make reſpecting the utility of a plant, of which he ſent the ſeed to this iſland, and which is ſaid to poſſeſs the virtues requiſite to cure the diſeaſe, and to call the attention of the Faculty to ſuch experiments as may aſcertain its real virtues and uſes in practice.

An account of this plant, called Zezegery, was lately publiſhed in a Letter from Captain Huxley to Sir Simon Clarke.

There is no doubt of its being the plant deſcribed by Brown, in his natural Hiſtory of Jamaica, p. 270, under the article Sesamum. *Foliis inferioribus trifidis dentatis, superioribus oblongis serratis*, commonly called Vanglo, or Oil Plant. It was introduced into the iſland by the Jews from Carolina; they valued it on account of the delicate oil contained in the ſeeds, which are ſaid to yield near one third of their weight in pure oil, by preſſure. The leaves being ſlightly bruiſed and immerſed in ſimple cold water, impart with ſurpriſing facility the mucilage, and in in great quantity; it appears perfectly limpid, without either smell or taste. In this state it was fit for use, it being made as thick as is agreeable to the patient.

Another species is described by the same author: *Foliis omnibus oblongis serratis*, which I have also seen; it possesses the mucilaginous quality of the leaves also.

Fortunately, the plant has been cultivated with considerable care by some Gentlemen here in their gardens, which afforded me an ample supply when the disease attacked the soldiers of the 55th regiment stationed at the Barracks.

The symptoms were acute, and I did not omit the remedies in general resorted to in this dangerous malady, but used the infusion of the Sesamum as common drink, and I thought with marked good effect. To be convinced whether its effects really assisted the cure, I omitted its use for a time, when the patients eagerly requested its renewal, and with declared advantage. The number of patients among the soldiers exceeds thirty, and we have met with no loss.

I requested the attention of some of the most respectable inhabitants, who were attacked with the disease, to its effects, whose reports are equally favourable; and what observations I have made in the negro practice, corroborate the idea of its being at least a most useful auxiliary. It certainly is one of the most bland and delicate mucilages I have met with; but, as was before hinted, I have not ventured to rely on its effect more than an auxiliary. This must be left to time, and probably *necessity*, when no other remedy may be at hand.

I am, &c.

*Lucea, Aug.* 20, 1802. GEORGE SPENCE.

